# Evaluating migraine with typical aura with neuroimaging

**DOI:** 10.3389/fnhum.2023.1112790

**Published:** 2023-03-21

**Authors:** Nazia Karsan, Elisa Silva, Peter J. Goadsby

**Affiliations:** ^1^Headache Group, School of Neuroscience, Wolfson Centre for Age-Related Diseases, Institute of Psychiatry, Psychology and Neuroscience, King’s College London, London, United Kingdom; ^2^NIHR King’s Clinical Research Facility, King’s College London, London, United Kingdom; ^3^Department of Neurology, University of California, Los Angeles, Los Angeles, CA, United States

**Keywords:** migraine, aura, visual aura, sensory aura, dysphasic aura, neuroimaging, fMRI, MRI

## Abstract

**Objective:**

To provide an up-to-date narrative literature review of imaging in migraine with typical aura, as a means to understand better migraine subtypes and aura biology.

**Background:**

Characterizing subtypes of migraine with typical aura and appreciating possible biological differences between migraine with and without aura, are important to understanding the neurobiology of aura and trying to advance personalized therapeutics in this area through imaging biomarkers. One means of doing this over recent years has been the use of increasingly advanced neuroimaging techniques.

**Methods:**

We conducted a literature review of neuroimaging studies in migraine with aura, using a PubMed search for terms ‘imaging migraine’, ‘aura imaging’, ‘migraine with aura imaging’, ‘migraine functional imaging’ and ‘migraine structural imaging’. We collated the findings of the main studies, excluding small case reports and series with *n* < 6, and have summarized these and their implications for better understanding of aura mechanisms.

**Results:**

Aura is likely mediated by widespread brain dysfunction in areas involving, but not limited to, visual cortex, somatosensory and insular cortex, and thalamus. Higher brain excitability in response to sensory stimulation and altered resting-state functional connectivity in migraine sufferers with aura could have a genetic component. Pure visual aura compared to visual aura with other sensory or speech symptoms as well, may involve different functional reorganization of brain networks and additional mitochondrial dysfunction mediating more aura symptoms.

**Conclusion:**

There is a suggestion of at least some distinct neurobiological differences between migraine with and without aura, despite the shared phenotypic similarity in headache and other migraine-associated symptoms. It is clear from the vast majority of aura phenotypes being visual that there is a particular predisposition of the occipital cortex to aura mechanisms. Why this is the case, along with the relationships between cortical spreading depression and headache, and the reasons why aura does not consistently present in affected individuals, are all important research questions for the future.

## Introduction

Whilst animal model and other experimental laboratory techniques have been valuable in studying migraine mechanisms, studying the multiple facets of the human disorder in humans is necessary to truly understand migraine and its related disability. The clinical phenotype of migraine in adults and in children and adolescents has evolved over time, and the International Classification of Headache Disorders (ICHD) has advanced with its different iterations to reflect this (Headache Classification Committee of the International Headache Society, 1988; Headache Classification Subcommittee of the International Headache Society, 2004; Headache Classification Committee of the International Headache Society [IHS], 2013, 2018).

Migraine aura is defined as ‘*recurrent unilateral visual, sensory or other central nervous system symptoms lasting minutes, that usually develop gradually and are usually followed by headache and associated migraine symptoms’* (Headache Classification Committee of the International Headache Society [IHS], 2018). As well as aura as an accompanying symptom to headache in migraine, it is important to acknowledge other non-painful manifestations of the attack as part of the clinical phenotype, distinct from aura. The current version of the classification (ICHD3) (Headache Classification Committee of the International Headache Society [IHS], 2018) contains reference to premonitory symptoms (PS) which start ahead of pain and are clinically heterogenous, as well as vestibular migraine, in which vertigo is a prominent feature. Several childhood syndromes and symptoms may be early markers of migraine, and suggest that the disorder can manifest in different ways at different stages of brain and biological development (Gelfand, 2015). There is increasing evidence for the presence of cranial autonomic symptoms (CAS) in migraine as well as in the trigeminal autonomic cephalalgias (TAC’s) (in which these symptoms are canonical), and additional symptoms are being recognized as CAS (Gupta and Bhatia, 2007; Lai et al., 2009; Gelfand et al., 2013; Guven et al., 2013; Raieli et al., 2015; Shin et al., 2015; Barbanti et al., 2016; Riesco et al., 2016; Danno et al., 2020; Karsan et al., 2022). It has not been possible within ICHD3 to subtype every different manifestation of migraine, rather to acknowledge that some symptoms like PS and CAS can occur in addition to the so-called canonical migraine symptoms; movement sensitivity, photophobia, phonophobia and nausea or vomiting (Headache Classification Committee of the International Headache Society [IHS], 2018).

The main subtypes of migraine identified within the classification are migraine with (1.2) and without (1.1) aura (Headache Classification Committee of the International Headache Society [IHS], 2018). Migraine with typical aura (1.2.1) is the focus of this review, and is a type of migraine with aura, involving visual, sensory or speech symptoms without motor weakness (Headache Classification Committee of the International Headache Society [IHS], 2018). Aura when present is most commonly visual in phenotype (Viana et al., 2017). Whilst ICHD3 claims the aura duration should be less than an hour in typical aura, and occur preceding or associated with headache, it is clear in clinical practice, aura more prolonged than an hour can be reported by patients (Viana et al., 2018), and that aura can occur at any time during the course of the migraine attack (Viana et al., 2016). It can also be persistent (although perhaps for less than a week), particularly in chronic migraine (Schankin et al., 2017). Persistent aura without infarction lasting over a week is defined separately in ICHD3 (1.4.2), as is typical aura without headache (1.2.1.2) (Headache Classification Committee of the International Headache Society [IHS], 2018). Typical aura in ICHD3 does not include hemiplegic or brainstem aura and these aura subtypes will therefore not be discussed in this review.

Aura attacks in migraine present in around a third of sufferers and clinically most often present initially as visual disturbances (Russell and Olesen, 1996). These disturbances can take a variety of forms and severities, including flashes of light, zig zag lines and loss of visual field (positive or negative symptoms). In a subgroup of patients, sensory (typically paraesthesiae) or language (paraphasia or aphasia) disturbances can either co-exist with visual aura or occur in isolation. These are likely mediated by the consecutive and successive involvement of different cortical areas beyond the occipital cortex in some individuals. The visual, sensory and speech symptoms in the typical migraine with aura subtype are thought to be mediated cortically, by involvement of the relevant part of the cerebral cortex. It is thought that the neurophysiological correlate of aura is a wave of spreading depolarization and subsequent repolarization called cortical spreading depression (CSD), first described by Leao (1944) in the rabbit cortex. Given this is a neurophysiological phenomenon, it has been largely observed in animal models rather than in the human disease, but increasing imaging evidence in humans has contributed to this being a feasible mechanism in aura (Olesen et al., 1981; Lauritzen et al., 1982, 1983; Lauritzen and Olesen, 1984; Cao et al., 1999; Hadjikhani et al., 2001), although ictal electroencephalography (EEG) studies in humans are lacking (Ogunyemi, 1995). A contributor to the clinical and biological heterogeneity of migraine is that different aura symptoms can co-exist in the same individual and can occur together simultaneously or in quick succession during the same attack (Viana et al., 2016). What mediates this propensity to aura in some individuals and why the occipital cortex is most likely to be involved in this phenomenon are areas that remain poorly understood.

There is no consensus about the relationship, or lack thereof, between CSD and trigeminal nociception (Borgdorff, 2018). Given the high frequency of aura and complexity of aura phenotypes in the monogenic genetic forms of migraine (Tolner et al., 2015), there is likely to be a strong genetic basis to the propensity to aura, although in most cases this cannot be attributed to a single gene. Aura can occur in the absence of headache and can also occur in different conditions. Most migraine with aura sufferers will experience more attacks of migraine without aura than with aura and the majority of migraine sufferers have similar headache and other associated symptom phenotypes whether they experience aura or not. These issues raise important questions as to whether CSD and migraine headache are directly related or are separate mechanisms entirely. Understanding this clinical and biological heterogeneity in migraine is vital to developing disease biomarkers and therapeutics, in an area where despite the current evolution of novel treatment options for migraine, there remains limited evidence for efficacy differences of migraine treatments between different migraine subtypes, and indeed a scarcity of treatments available for those in whom the aura itself is particularly problematic, due to severity, frequency, duration or persistence, thus leaving an underserved population of patients.

Many insights into understanding aura mechanisms have come from animal models of CSD (Harriott et al., 2019) and interictal human neurophysiological studies (Coppola et al., 2019). In recent times, the development of functional neuroimaging methods and the ability to study the human migraine attack, at times ictally, and mostly interictally, and its symptoms phenotypically, have advanced our understanding of the neural substrates for migraine aura and alluded to therapeutic substrates that may be explored for aura treatment. There seems to be a predisposition to cortical excitability and sensory hypersensitivity in migraine with aura, which is enhanced when compared to migraine without aura (Demarquay and Mauguière, 2016). As visual aura is the most common aura subtype, even in those who may also have other aura symptoms, it has been the most frequently studied. Sensory and speech aura (as well as motor, hemiplegic and brainstem aura) have also been studied to a lesser extent. This review will summarize the current imaging literature in migraine with typical aura and the insights that these studies and imaging methodologies have provided into the neurobiology of aura and its subtypes.

## Search strategy

This was not formulated to be a systematic review, more a summary of some key findings of imaging studies in this area. A search was conducted of the PubMed database between October and December 2022. The search terms were “migraine” AND “aura” AND (“neuroimaging” OR “neuro-imaging” OR “structural” OR “functional”). In addition, review articles and the reference lists of the included articles were also checked to identify potential omitted studies in the search. Case reports and case series with less than 6 subjects were excluded based on the vastness of the literature in this area. There is one exception to this, which is a landmark study by Hadjikhani et al. (2001), which managed to capture ictal aura and was the first to demonstrate real time alterations in the visual cortex, likely correlating with CSD mechanisms previously only demonstrated neurophysiologically in animal models.

Findings from the remaining studies were collated into the appropriate tables and text within this review.

## Means of imaging aura

### Clinical imaging

In the clinical setting, imaging with computerized tomography (CT) and magnetic resonance imaging (MRI) is usually performed if a patient presents with their first aura, change in aura phenotype or prolonged aura. Migraine aura is a common clinical stroke mimic, when presenting for the first time so it is standard to have urgent CT or MRI performed acutely in these situations. A non-contract CT of the head is expected to be normal in migraine with aura (Ridolfi et al., 2018), as are conventional MR sequences (Hougaard et al., 2017a).

#### Ictal or peri-ictal imaging

In the ictal or perictal phase, it is not uncommon for reversible transient diffusion restriction (Bereczki et al., 2008; Parks et al., 2014) to be observed during aura, and during a prolonged period of oligemia, migrainous infarction has also been reported (Serrano et al., 2018). Many of these ictal or peri-ictal studies are small, owing to the challenges in capturing spontaneous aura with imaging, and in triggering aura experimentally. Whilst prolonged oligemia can cause tissue infarction, one study has suggested that flow rates to involved brain tissue during migraine aura are higher than those when compared to stroke (Förster et al., 2014). Some case reports of reversible lesions of the corpus callosum in patients with migraine with typical aura exist (Lin and Yang, 2011; Lewis et al., 2020), with these lesions likely resulting from the high vulnerability of this area to non-specific cytotoxic damage. Such damage is felt to be likely mediated by glutamate, and callosal changes have also been demonstrated in migraine in general (Tantik Pak et al., 2022).

During CSD, neurovascular coupling is disturbed and the increase in tissue demand is accompanied by a reduction in arterial supply and therefore an increase in deoxygenated hemoglobin. This change can be exploited with susceptibility weighted MR imaging (SWI) or T2* gradient echo sequences on MRI, both of which are routinely available in many centers, in particular those managing hyperacute and acute stroke (Kellner-Weldon et al., 2021). These MR techniques can ictally show engorgement of veins, prominent focal veins (PFV), and this finding is more frequently documented less than 24 h from the onset of symptoms, with a possible increase in oxygen extraction in areas where the CSD wave has passed (Kellner-Weldon et al., 2021). Various studies over recent years have utilized this technique and postulated the possible acute clinical use in the emergency setting for differentiating migraine aura from other diagnostic mimics (Breiding et al., 2020; Kellner-Weldon et al., 2020, 2021; Slavova et al., 2020; Hirtz et al., 2022). The findings of these studies are included in Table 1.

**TABLE 1:** Summary of structural imaging studies in migraine with typical aura.

References	Subjects	Imaging phase	Imaging modality	Key findings	Translation of findings
Matharu et al., 2003	11 MwA 11 HC 17 MwoA 17 HC	Interictal	T1-weighted MRI	No significant difference in global gray or white matter volumes between either patients with migraines and controls, or patients with aura and without aura	No macroscopic structural differences between migraine and controls and in those with aura
Granziera et al., 2006	12 MwA (visual) 12 MwoA 15 HC	Interictal	T1-weighted MRI, DTI	Increases in cortical thickness in motion-processing visual areas (middle temporal and V3A) in MwA and MwoA relative to HC with changes in superior colliculus (SC) and lateral geniculate nucleus (LGN)	Changes in V3A (which is thought to be the origin of CSD) and visual processing may occur in both MwA and MwoA and mediate asymptomatic aura in MwoA
DaSilva et al., 2007b	12 MwA (8 pure visual and 4 visual and sensory) 12 MwoA 12 HC	Interictal	T1-weighted MRI, DTI and fractional anisotropy (FA)	Lower FA in ventral trigeminothalamic tract in MwA compared to without, lower FA of ventrolateral PAG in MwoA	Additional alterations in sensory processing in MwA
Rocca et al., 2008	7 MwA (visual) 8 MwoA	Interictal	DTI	Optic radiation changes in MwA not correlating to clinical or structural metrics	Optic radiation diffusivity changes could be a biomarker for MwA
Kruit et al., 2010	161 MwA 134 MwoA 140 HC	Interictal	T2 and FLAIR MRI	Subjects with migraine, especially MwA had a higher prevalence of subclinical posterior infarcts (OR 13.7)	Aura may be an independent risk factor for stroke
Yilmaz-Kusbeci et al., 2010	23 MwA 24 HC	Interictal	T1-weighted MRI	No differences in cerebellar or cerebral volumes or ratios between groups	No differences in cortical or cerebellar volume in MwA
Cucchiara et al., 2013	56 MwA 61 MwoA 53 HC	Interictal	MRA	Migraine groups were more likely to have an incomplete circle of Willis compared to controls, with associated hemispheric hypoperfusion in the PCA territory, with an increased rate of variants in the aura group	MwA is more likely to be associated with an incomplete circle of Willis and corresponding differences in cerebral blood flow
Granziera et al., 2014	22 MwoA 15 MwA (visual) 20 HC	Interictal	T1 and T2*-weighted MRI, diffusion spectrum imaging	Microstructural broad thalamic alterations in MwA suggesting increased iron deposition and myelin cellularity	These changes may mediate increased cortical excitability in MwA
Hougaard et al., 2015a	20 MwA (visual, side-locked)	Interictal	T1-weighted MRI	No differences in gray matter structure with regards to aura symptoms in MwA patients. Reduced cortical thickness in inferior frontal gyrus in headache hemisphere compared to contralateral	No gray matter structural changes in MwA. Inferior frontal cortex structural changes suggest structural reorganization of pain inhibitory circuits in response to migraine attacks
Hougaard et al., 2016	60 MwA (visual ± sensory) 60 HC	Interictal	T1-weighted MRI	No differences in gray matter or cortical structure between groups	Aura is not caused by and does not cause structural brain differences
Gaist et al., 2018	166 MwA (98% visual) 30 co-twins 137 unrelated HC	Interictal	T1-weighted MRI	Thicker visual cortex (V2 and V3A) in MwA compared to HC and in V2 compared to discordant co-twins, with no relationship to aura frequency	Cortical thickness changes in females with MwA may be an inherent genetic trait and independent of subsequent aura frequency
Petrusic et al., 2018	48 MwA (visual and visual + sensory and dysphasia) 30 HC	Interictal	T1 and T2-weighted MRI	Complex aura group had reduced cortical surface area of the left rostral middle frontal cortex compared with pure visual aura group. Migraine patients had reduced volume of the left fusiform gyrus relative to HC. Sulcal depth increased at the level of the left temporal pole in the complex aura group relative to the pure visual aura group	MwA demonstrates different morphometric features from HC in multiple cortical regions. Complex aura involves different morphometric features in the left frontal and temporal lobe relative to pure visual aura, which could mediate symptoms and form an imaging biomarker for different aura types
Khouri Chalouhi et al., 2018	47 MwA	Interictal	T1 and T2-weighted, fluid attenuated inversion recovery (FLAIR), diffusion-weighted MRI and T2 double inversion recovery (DIR)	4% of those with aura (visual) had cortical abnormalities, in left-sided frontal and cingulate cortex	CSD does not cause structural cortical change and is a transient event without structural sequelae
Zhang et al., 2018	52 MwA 58 MwoA 52 HC	Interictal	MRA, ASL MRI and FLAIR	Greater displacement of basilar artery in MwA compared to MwoA and HC, increasing with age and baseline headache frequency without associated perfusion change	Basilar artery displacement may be associated with aura and disease activity
Magon et al., 2019	38 MwA 93 MwoA 115 HC	Interictal	T1-weighted MRI	Thinner cortex in subparietal sulcus bilaterally, left intraparietal sulcus and right anterior cingulate in MwA compared to HC	Different structural brain abnormalities in MwA compared to MwoA, perhaps caused by different genetic disease mechanisms and effect of repeated attacks on brain structure
Petrusic et al., 2019a	32 MwA (undifferentiated) 32 HC	Interictal	T1 and T2-weighted MRI	Smaller globus pallidus and putamen volumes in MwA compared to HC	The basal ganglia may be involved in MwA
Petrusic et al., 2019b	42 MwA 42 HC	Interictal	T1 and T2-weighted MRI	Larger brainstem volume in MwA compared to HC without clinical correlation with aura phenotype	The brainstem may be involved in MwA
Bonanno et al., 2020	14 MwA (visual) 14 MwoA 14 HC	Ictal	T1-weighted MRI	Different global gray matter volume between all migraine groups and HC. MwA had reduced gray matter volume in 4 regions compared to HC (right cerebellum, left postcentral and precentral gyrus, right inferior frontal gyrus, left Broadman area 20–22 and left lingual gyrus), and more gray matter volume in right superior parietal gyrus and left thalamus compared to reduced gray matter volume in bilateral cerebellum, left cerebellum crus I, left superior/medial and right inferior/middle frontal gyrus, right superior frontal gyrus, left fusiform gyrus, left Broadman area 20, right parahippocampal gyrus and insula in and increased in right thalamus in MwoA compared to HC	Migraine with and without aura demonstrate different interictal structural brain differences compared to HC
Hougaard et al., 2020	156 MwA 120 HC 29 migraine aura free co-twins	Interictal	T1 and T2-weighted MRI, FLAIR	No differences in thalamic volume between groups or differences in individual thalamic nuclei	Thalamic volume alterations are not involved in mediating MwA
Breiding et al., 2020	50 MwA	Within 24 hours of symptoms	SWI MRI	Visual venous asymmetry present in 40%, with prominent focal veins laterality correlating with the laterality of these findings. The average time between aura onset and the imaging being performed correlated negatively with the venous volume of the dominant hemisphere	Unilateral hemispheric deoxygenation may be an early imaging sign of MwA
Kellner-Weldon et al., 2020	99 MwA (children and adolescents; sensory, speech and visual and less commonly weakness and confusion)	Within 24 hours of symptom onset in 62.6%	SWI MRI	Focally increased deoxygenation in 61.6% (left- hemispheric dominance) correlating with contralateral aura symptoms, some with increased perfusion in corresponding regions (mainly temporal, occipital and fronto-parietal)	SWI abnormalities in MwA are common and occur with a lateralized dominance although can be bilateral
Slavova et al., 2020	6 MwA	Ictal	SWI MRI	Accentuated sulcal vein pointing towards cortical area of relevance in mediating aura symptoms (exceeding corresponding contralateral vessel by a mean factor of 2)	SWI acutely may be a useful tool in the emergency evaluation of migraine with aura
Özkan and Gürsoy-Özdemir, 2021	21 MwA (25% visual) 63 MwoA	Interictal	T1-weighted MRI	Significantly higher rates of occipital bending in migraine with visual aura compared to MwoA	Increased rates of occipital bending may be implicated in mediating visual aura
Kellner-Weldon et al., 2021	638 MwA (visual, sensory, speech and less frequently motor)	Within 5 days of symptoms	SWI MRI	Abnormal imaging in 18.8%, with temporal and occipital lobes most commonly affected contralateral to aura symptoms, more likely to be abnormal if performed within 24 hours of symptom onset	Prominent focal veins on SWI can be used as an acute imaging marker of MwA
Hirtz et al., 2022	60 diagnosed MwA or probable MwA (various but prolonged and severe and non-motor) 60 non-migrainous controls with neurological symptoms with no structural lesion	Within 24 hours of symptom onset	T2* gradient echo	Cortical vein sign (marked hypo-intensity and/or an apparent increased diameter of at least one cortical vein) in 42% of MwA compared to none in HC group, predominantly bilaterally and posteriorly, associated with increased severity of aura symptoms and shorter time delay to MRI	Acute MRI imaging of aura with a T2* sequence can reveal a reliable cortical vein sign in MwA

MwA, migraine with aura; MwoA, migraine without aura; HC, healthy controls; VBM, voxel-based morphometry; MRI, magnetic resonance imaging; DTI, diffusion tensor imaging; SC, superior colliculus; LGN, lateral geniculate nucleus; CSD, cortical spreading depression; PAG, periaqueductal gray; MRA, magnetic resonance angiography; PCA, posterior cerebral artery; ASL, arterial spin labeling; SWI, susceptibility-weighted imaging.

#### Interictal imaging

A new structural technique which has been used, is that of assessing occipital bending on structural MR sequences in migraine with aura patients (Özkan and Gürsoy-Özdemir, 2021). This is a human anatomical asymmetrical variant in which one occipital pole crosses the midline and bends over the contralateral pole. This anatomical variant has been thought to be three times more common in those with certain psychiatric disorders including major depression (Maller et al., 2014), bipolar affective disorder (Maller et al., 2015) and schizophrenia (Maller et al., 2017). Given only one study to our knowledge has been conducted using this technique in migraine, further studies assessing the reliability of this sign are awaited and this is not a widely used clinical technique at the moment.

Abnormalities on MR angiography (MRA) sequences have also been described in migraine with aura; one study suggests that in an incomplete circle of Willis is more common in migraine than healthy controls, with an increased risk of variants in those with aura (Cucchiara et al., 2015), and another hypothesizing that lateral basilar artery displacement is a feature interictally in migraine with aura (Zhang et al., 2018). Whilst MRA is not routinely used to evaluate aura, it may be performed as part of a workup for possible stroke, and further systematic studies looking into possible interictal arterial changes in aura are warranted. The two available studies do suggest that there may be alterations to posterior circulation vasculature in migraine with aura with one of these studies suggesting associated perfusion changes (Cucchiara et al., 2015) and one not (Zhang et al., 2018).

### Structural imaging

#### Volumetric and cortical thickness changes

Advanced macro and microstructural imaging techniques, such as voxel-based morphometry (VBM) and diffusion tensor imaging (DTI), have allowed detailed comparison of interictal brain structure between migraine with and without aura and comparison to healthy controls. Increased cortical thickness in the V3A area of visual cortex has been demonstrated in both migraine with and without aura, suggesting that extrastriate visual areas are implicated in both migraine subtypes, irrespective of aura symptoms being present or not (Granziera et al., 2006). This study postulated that asymptomatic or silent CSD could be a phenomenon in those with migraine without aura clinically.

Further studies have confirmed findings of increased cortical thickness in visual cortical areas in migraine with aura compared to healthy controls and co-twins without aura (Gaist et al., 2018). Other studies have demonstrated differences in cortical thickness in areas outside of visual cortex in subjects with migraine (with and without aura) compared to healthy controls, involving somatosensory cortex (DaSilva et al., 2007a,b), executive and visual cortical areas (Messina et al., 2013) and inferior frontal gyrus (Hougaard et al., 2015a). Whilst it is perhaps unsurprising that brain areas that could feasibly be mediating additional aura symptoms and those that are involved in other pain processing and modulation may be involved in migraine with aura, it is difficult to know if such changes are the result of the condition or are a predisposition to the condition. There have been some inconsistences between studies, with some studies failing to replicate structural findings and instead demonstrating no macrostructural differences between migraine with and without aura and when compared to healthy controls (Datta et al., 2011; Hougaard et al., 2016). Given the variability in findings in these studies in migraine with aura, and similar findings in other studies between migraine with and without aura, it is difficult to conclude whether there are fundamental gray matter structural differences between migraine with and without aura and compared to healthy controls. A meta-analysis also failed to provide consistency of findings for gray matter volume differences in migraine compared to controls in studies using VBM (although migraine with aura was not sub-analyzed) (Mehnert et al., 2020). Nine studies using either VBM or DTI were examined in a meta-analysis by Bashir et al. (2013), and the authors found that five studies reported reductions in gray matter volume associated with increasing attack frequency and disease duration. One study reported increased periaqueductal gray and dorsolateral pons volume in migraine with aura compared to migraine without aura, and these findings have also been supported by another study (Petrusic et al., 2019b).

A study by Hougaard et al. (2015a) studied the laterality of structural gray matter changes and compared cortical thickness in 20 subjects with side-locked visual aura with their migraine attacks. The authors demonstrated no gray matter structural changes but reduced inferior frontal gryus cortical thickness on the headache hemisphere compared to the contralateral hemisphere and suggested that alterations in frontal cortex structure are a result of repeated migraine attacks altering pain modulation circuits. Hougaard et al. (2016) also demonstrated that whilst gray matter volume in the anterior cingulate was reduced in migraine with aura relative to controls, there was no difference in gray matter volume or cortical thickness in those with and without sensory aura. A further study by Petrusic et al. (2018) found differences in brain structure when comparing pure visual aura to visual aura with sensory and dysphasic symptoms and suggested that the additional findings in this group could serve as an imaging biomarker for distinguishing aura subtypes.

Other studies have specifically examined subcortical nuclei volumes between migraine with and without aura and compared to healthy controls, again with conflicting results. One study demonstrated broad thalamic structure alterations in migraine with aura (Granziera et al., 2014), and another failed to replicate these findings (Hougaard et al., 2020). Another study suggested other deep brain nuclei differences in the putamen and globus pallidus in migraine with aura (Petrusic et al., 2019a), with reduced volumes but larger brainstem volumes in migraine with aura compared to healthy controls (Petrusic et al., 2019b), suggesting that deep subcortical brain nuclei and brainstem structures may be altered in migraine with aura. The influence of disease activity, that is underlying headache frequency and disease duration, on structural imaging findings in migraine is unclear as various studies have reported conflicting findings (Sheng et al., 2020, 2021).

A meta-analysis of VBM studies in migraine published recently suggested a range of findings in migraine in general compared to healthy controls, with areas involved in sensory, cognitive, pain and affective processing being implicated. Their pooled analysis of migraine subtypes suggested distinct patterns of gray matter volume change between migraine with aura (more occipital and temporal involvement) and migraine without aura (Zhang et al., 2023).

#### White matter hyperintensities

White matter disease has also been evaluated in migraine given early observations of an increase in white matter disease burden on brain imaging in patients with migraine (Igarashi et al., 1991). These observations led to a large population-based study called the CAMERA study, which evaluated large groups of migraine with and without aura and healthy controls and compared baseline interictal MR brain imaging between the groups (Kruit et al., 2004). The study demonstrated that women with migraine are more likely to have supratentorial white matter hyperintensities, with the number correlating with baseline attack frequency. A sub-analysis demonstrated that this was more common in those with migraine with aura, particularly in the posterior circulation (Kruit et al., 2010). A subsequent 9-year follow up study (CAMERA 2) showed no other progressive structural brain changes and no cognitive impact of the white matter hyperintensities (Palm-Meinders et al., 2012). A subsequent meta-analysis of 19 studies has confirmed the suggestion of an increased risk of white matter hyperintensities in migraine compared to healthy controls, with a particular risk in those with aura, without an association in this group with silent brain infarcts (Bashir et al., 2013).

The mechanisms behind these white matter changes are unknown but have been hypothesized as being related to cerebral blood flow changes (Kruit et al., 2010; Zhang et al., 2017), arterial narrowing (Kruit et al., 2005), as well as the presence of a patent foramen ovale (PFO) (Yeo et al., 2022) and blood brain barrier disruption (Albrecht et al., 2019), among others. As the majority of patients with migraine with aura do not have a PFO (although it is 2-3 times more common in migraine without aura compared to the normal population (Caputi et al., 2009; Liu et al., 2020), and other imaging studies have suggested an intact blood brain barrier during attacks of migraine with (Hougaard et al., 2017a) and without aura (Amin et al., 2017), the presence of these lesions is likely to be multifactorial. In particular, age and cardiovascular risk factors are likely to play a role, and these have not been evaluated in subgroup analyses of the population data from CAMERA (Kruit et al., 2005).

Whilst the studies discussed have yielded conflicting results (Masson et al., 2021), it is possible that fundamental structural differences in migraine with aura involving areas like visual and extrastriate cortex, are likely mediating the disease process and clinical phenotypes. These may be caused by an underlying genetic predisposition to migraine with aura differentially affecting these brain regions, rather than being influenced by underlying attack frequency or disease activity. Interestingly, there does not seem to be a clinical correlation between structural brain areas involved and clinical aura phenotypes. It is clear that on a population level, younger women with migraine with aura are more likely to have white matter hyperintensities on structural brain imaging which are largely posterior and supratentorial, correlating with baseline attack frequency and disease duration, and not associated with progressive cognitive impairment at follow up.

Some of the main structural brain imaging studies evaluating migraine with aura are summarized in Table 1.

### Perfusion studies

Several perfusion imaging modalities have been used for research purposes to evaluate migraine with aura. Most are not available for wide scale clinical use. This is an obvious choice of imaging methodology in aura, given the vascular and perfusion changes likely to occur with CSD and alterations in neurovascular coupling, which can be exploited using perfusion imaging methods. The potential utility of such techniques if available in clinical settings for differentiating migraine aura from stroke, is also attractive. These techniques have included historical intra-arterial Xenon and have evolved to include single photon emission computed tomography (SPECT) and advanced forms of perfusion MRI.

#### Ictal or peri-ictal imaging

Initial intra-arterial Xenon techniques used by Lauritzen et al. (1983), Olesen et al. (1990) were the first to show objective alterations in regional cerebral blood flow (rCBF) in migraine aura during triggered attacks occurring posteriorly, and propagating at a rate compatible with CSD. These studies provided evidence for CSD likely mediating aura in human subjects. Since then, more advanced non-invasive MR options have become available as a means of assessing cerebral perfusion without the need for ionizing radiation and contrast material. MR perfusion-weighted imaging or arterial spin labeling (ASL) (Sanchez del Rio et al., 1999; Jäger et al., 2005; Arkink et al., 2012; Förster et al., 2014; Cadiot et al., 2018; Kellner-Weldon et al., 2018; Uetani et al., 2018; Wolf et al., 2018; Michels et al., 2019; Fu et al., 2022), or indeed CT perfusion techniques (Gonzalez-Martinez et al., 2021) (this may be more likely to be available clinically), may show transient regional cerebral perfusion changes in aura, reflecting the hemodynamic variations occurring. Typically, such changes affect more than one vascular territory, can be bilateral, and tend to predominate posteriorly, but a variety of ictal and interictal perfusion changes have been demonstrated in migraine with aura using different imaging modalities and are summarized in Table 2.

**TABLE 2:** Summary of perfusion studies in migraine with typical aura.

References	Subjects	Imaging phase	Imaging modality	Main findings	Translation of findings
Lauritzen et al., 1983	9 MwA (7 visual and sensory, 1 sensory and speech and 1 sensory)	Ictal (angiography triggered MwA)	Intra-arterial Xenon	89% had reductions in rCBF, with spreading oligaemia at 2mm/min and stopped by primary brain sulci. These changes were observed posteriorly and propagated anteriorly to parietal and temporal lobes. These occurred before symptoms and outlasted focal symptoms before and outlasting focal symptoms	Likely relationship between migraine aura and Leao’s spreading depression
Olesen et al., 1990	63 MwA (various)	Ictal (triggered)	Intra-arterial Xenon, inhaled Xenon and SPECT	Unilateral reductions in rCBF can precede aura and headache can occur during the hypoperfusion but hyperperfusion during this time can also occur. Aura symptoms and headache are usually contralateral but unilateral hemispheric changes can cause bilateral headache	Cerebral hypoperfusion precedes aura and occurs during aura, and can occur during headache, when hyperperfusion can also occur. Aura symptoms are mediated unilaterally and perfusion changes tend to persist unilaterally even if headache is bilateral
Soriani et al., 1997	30 MwA	19 ictal 11 interictal	SPECT	Regional cerebral blood flow (rCBF) abnormalities in 74%; hypoperfusion in 11/14 patients and hyperperfusion in 3/14 patients. In most cases, rCBF abnormalities corresponded to neurological symptoms	Corresponding perfusion changes occur during MwA and usually involve hypoperfusion
Sanchez del Rio et al., 1999	6 aura phase MwA (visual) 3 headache phase MwA 13 MwoA	Ictal	Contrast perfusion MRI	Perfusion deficits in contralateral occipital cortex to field defect in all 7 subjects imaged during aura, persistent during headache despite resolved aura in two subjects imaged during subsequent headache (but peak change during aura). No interictal changes and no perfusion changes in MwoA group	MwA is associated with contralateral cortical hypoperfusion which can persist during headache, even if headache is ipsilateral
Jäger et al., 2005	2 MwA (persistent visual) 2 visual snow syndrome	Ictal	Contrast perfusion MRI and MR diffusion	No differences in water diffusion or perfusion in occipital cortex or other brain regions between the two groups	Water diffusion or perfusion defects do not play a role in mediating persistent visual aura or visual disturbances
Arkink et al., 2012	12 MwA 17 MwoA 16 HC	Interictal	Contrast perfusion MRI	Hypoperfusion in postcentral gyrus and inferior temporal gyrus in MwA and inferior frontal gyrus in MwoA	Different patterns of interictal perfusion in MwA and MwoA
Förster et al., 2014	33 MwA (any; sensory most common) 33 age-matched ischaemic stroke	Within 6 hours of symptom onset	Contrast perfusion MRI	Hypoperfusion in 54.5% of MwA (compared to 60.6% of those with stroke), without DWI lesions. 61.1% of the MwA ones had occipital predominance and in 27.8% occipital and parietal without correlation with clinical presentation (usually involving two or more vascular territories and with normal MRA). The stroke ones were MCA/PCA territory with 9.1% involving more than one vascular territory with vessel stenosis or occlusion on MRA. Significantly lower MTT (mean transit time) and TTP (time to peak) in MwA compared to stroke	MwA usually associated with a perfusion deficit not limited to a specific vascular territory, and a moderate increase of TTP Hypoperfusion restricted to a single vascular territory with a marked increase of TTP and/or MTT is atypical for migraine with aura and suggestive of acute ischemic stroke Such differences could help in the acute diagnosis of aura as a stroke mimic
Hougaard et al., 2017a	19 MwA (visual, sensory, aphasia and mixed)	Ictal (headache) and interictal	Dynamic contrast-enhanced MRI	Intact blood brain barrier following attacks of MwA, hypoperfusion in the visual cortices and posterior white matter contralateral to perceived aura symptoms, increased bilateral lower pons perfusion during headache following aura	Contralateral cortical hypoperfusion persists following aura
Wolf et al., 2018	4 MwA (2 visual, 1 sensory and motor, 1 speech and motor)	2 during aura 2 during headache	ASL MRI	Right temporal hyperperfusion and right parieto-occipital hypoperfusion or hyperperfusion during aura which normalised on follow up	Reversible mixed perfusion changes during the aura period in more than one vascular territory
Cadiot et al., 2018	17 MwA (children; visual, speech, sensory and motor)	Within median 6 hours of aura onset	MRI, time of flight MRA and ASL MRI	Hypoperfusion in one or more cerebral lobes in 94% with vasospasm on MRA in 71%	Ictal hypoperfusion and associated vasospasm may be a useful marker of aura acutely
Uetani et al., 2018	8 MwA 41 MwoA (children and adolescents)	Within 7 days of symptoms	ASL MRI	22% had perfusion abnormalities, usually occipital in 73% and parietal in 64%, usually in multiple regions in 82%, more likely if imaged within 24 hours of symptom onset and if aura present (6/8 with aura had perfusion changes)	Aura may be associated with occipital hypoperfusion in children and adolescents within 24 hours of symptom onset
Kellner-Weldon et al., 2018	98 MwA (visual, sensory, motor, language)	Within 24 hours of aura onset	Contrast perfusion MRI	22% had hemispheric cortical hypoperfusion, with 52% of these having contralateral cerebellar hypoperfusion as well	Episodic and brief aura may be associated with non-ischaemic hypoperfusion of cortex and cerebellum as a benign phenomenon
Michels et al., 2019	11 MwA 6 MwoA 19 HC	Interictal	ASL MRI	Increased perfusion occipitotemporal regions in migraine compared to HC, with increased changes in MwA compared to MwoA	Interictal posterior perfusion changes may be a marker of MwA
Gonzalez-Martinez et al., 2021	25 MwA (mainly sensory, 3 aphasia)	Ictal (median 171 minutes since symptom onset)	Multimodal CT (CT, angiography and perfusion)	12% had hypoperfusion, pattern not restricted to a single vascular territory	Acute perfusion abnormalities not common in MwA but when present do not respect a single vascular territory
Fu et al., 2022	32 MwA (22 visual, 8 sensory, 4 speech) 56 MwoA 44 HC	Interictal	Pseudocontinuos arterial spin labeling (pCASL)	Higher CBF in MwA in superior frontal gyrus, postcentral gyrus, cerebellum and lower CBF in middle frontal gyrus, thalamus, and medioventral occipital cortex	Differences in interictal perfusion in MwA compared to MwoA and HC

MwA, migraine with aura; MwoA, migraine without aura; HC, healthy controls; rCBF, regional cerebral blood flow; SPECT, single-photon emission computed tomography; SWI, diffusion-weighted imaging; TTP, time to peak; MCA, middle cerebral artery; PCA, posterior cerebral artery; MTT, mean transit time; ASL, arterial spin labeling; MRA, magnetic resonance angiography; MRI, magnetic resonance imaging; CBF, cerebral blood flow.

In general, it seems that cerebral hypoperfusion in one or more vascular territories occurs mainly unilaterally early during aura in the ictal phase and persists throughout the duration of the aura and sometimes as headache starts, before hyperperfusion occurs. Headache can occur from the same hemisphere as the one mediating aura, even if headache is bilateral, suggesting that bihemispheric brain involvement is not needed for the perception of bilateral head pain. These perfusion changes are thought to be the imaging correlate for CSD in these subjects, as the affected brain region with perfusion change tends to correlate in terms of both laterality and anatomical location with the clinical aura phenotype. The hypoperfusion may be associated with radiological vasospasm on imaging. Imaging as close as possible to the time of onset of aura symptoms tends to yield the most positive results. The pattern of ictal perfusion change can help distinguish aura from stroke in terms of number of vascular territories involved, and ictal abnormalities on MRA can occur in migraine aura [vasoconstriction (Förster et al., 2014; Cadiot et al., 2018) or mild dilation (Förster et al., 2014)].

#### Interictal imaging

Interictal perfusion differences may exist between migraine with and without aura and involve a combination of hypo and hyperperfusion of different brain regions. These are also summarised in Table 2.

### Functional imaging

Functional imaging studies have provided contributory evidence to the perfusion literature and have suggested ictal and interictal alterations in functional connectivity within visual and extrastriate cortex (Cao et al., 1999, 2002; Datta et al., 2013; Hougaard et al., 2014; Niddam et al., 2016; Tedeschi et al., 2016; Arngrim et al., 2017, 2019; Russo et al., 2019; Silvestro et al., 2022), as well as in other areas such as limbic regions (Faragó et al., 2017), and sensory (Hougaard et al., 2017b) and executive cortical areas (Coppola et al., 2022), amongst other alterations in migraine with aura. These studies are summarized in Table 3.

**TABLE 3:** Summary of functional imaging studies in migraine with typical aura.

References	Subjects	Imaging phase	Imaging modality	Main findings	Translation of findings
Cao et al., 1999	10 MwA 2 MwoA 6 HC	Interictal but 2 triggered auras captured	fMRI	Suppression of initial activation preceding triggered visual symptoms and headache slowly propagated into contiguous occipital cortex at a 3 –6 mm/min, accompanied by contrast intensity increases suggesting vasodilatation and tissue hyperoxygenation	Visually triggered headache and visual change in MwA is accompanied by spreading suppression of initial neuronal activation and increased occipital cortex oxygenation. Spreading suppression may be associated with the initial activation of a migraine attack, independent of whether there are associated aura symptoms or not
Hadjikhani et al., 2001	3 MwA (visual)	Ictal (triggered)	fMRI	BOLD signal changes demonstrated at least eight characteristics of CSD, time-locked to percept/onset of the aura (initial increase in BOLD signal in extrastriate cortex (area V3A) progressing contiguously and slowly (3.5 ± 1.1 mm/min) over occipital cortex, congruent with the retinotopy of the visual percept, then BOLD signal diminished with BOLD response to visual activation	CSD generates human visual aura in the visual cortex
Cao et al., 2002	23 MwA 3 MwoA 10 HC	Interictal (but 10 had triggered aura by visual stimulation)	fMRI	75% of the patients who developed symptoms during stimulation (headache or visual), had increased signal intensities in red nucleus and substantia nigra before occipital cortex signal elevation and before visual symptom onset	Red nucleus and substantia nigra activation precedes visual cortex activation in MwA, suggesting a dysfunctional brainstem cortical network ahead of headache
Datta et al., 2013	25 MwA 25 MwoA 25 HC	Interictal	fMRI and ASL MRI	Increased BOLD responses in the visual cortex and LGN to visual stimulation in MwA compared to other groups	Interictal cortical hyperresponsiveness in visual networks in MwA
Hougaard et al., 2014	20 MwA (unilateral side-fixed visual) 20 HC	Interictal	fMRI	BOLD responses to visual stimulation increased in symptomatic hemisphere in MwA in parietal and frontal regions when compared to HC	MwA involves a lateralised hyperexcitable visual system interictally
Tessitore et al., 2015	20 MwA 20 MwoA 20 HC	Interictal	fMRI, VBM and DTI	Disrupted executive control network functional connectivity in both migraine groups compared to HC with no differences in MwA and MwoA groups, without functional deficit	No resting state functional connectivity differences in the executive control network between MwA and MwoA but abnormal findings in both groups compared to HC
Hougaard et al., 2015b	40 MwA (visual) 40 HC	Interictal	fMRI	No differences in intrinsic resting functional connectivity between groups in any brain regions of interest, including occipital cortex, amygdala and PAG	Ictal changes associated with aura are likely transient and not due to an inherent difference in resting brain activity in MwA
Tedeschi et al., 2016	20 MwA 20 MwoA 20 HC	Interictal	fMRI, VBM and DTI	Increased functional connectivity in MwA group between lingual gyrus and visual network without corresponding structural or microstructural change	Extrastriate cortical involvement in MwA which likely initiates and propagates CSD
Niddam et al., 2016	26 MwA 26 MwoA 26 HC	Interictal	fMRI	Reduced connectivity between anterior insular and occipital regions including V3A in MwA correlating with headache severity	Interictal alterations in functional connectivity within the visual networks likely predisposes to aura
Arngrim et al., 2017	5 MwA	Ictal (triggered aura)	fMRI	Reduced occipital cortex BOLD response to visual stimulation during negative visual aura symptoms and increased response in positive visual symptoms, bilateral symptoms were associated with hemispheric changes	Correlation of BOLD signal and clinical aura phenotype CSD can occur bilaterally
Faragó et al., 2017	18 MwA (17 visual, 1 sensory) 35 MwoA 32 HC	Interictal	fMRI	Resting state BOLD fluctuation higher in MwA in cingulate cortex, superior parietal lobule, cerebellum and bilateral frontal regions compared to MwoA with increased amplitude of activity in MwA in all resting state networks	The cortex is more hyperexcitable in MwA compared to MwoA and HC
Lo Buono et al., 2017	14 MwA 14 MwoA 14 HC	Interictal	fMRI	Increased connectivity in left angular and supramarginal gryi, right precentral and postcentral gryi and right insular cortex in MwA compared to HC and MwoA	Altered functional architecture in MwA which could allude to pathophysiological mechanisms
Hougaard et al., 2017a	16 MwA (visual)	Ictal and interictal	fMRI	Increased connectivity ictally between left pons and somatosensory cortex and between V5 and middle frontal gyrus	Ictal alterations in functional connectivity during aura may link the aura symptoms and headache
Arngrim et al., 2019	15 MwA 14 HC	Interictal during exposure to hypoxia but 8/15 had aura attacks triggered by hypoxia and not sham gas	fMRI	Hypoxia induced greater BOLD response to visual stimulation in MwA compared to HC in the visual cortex	MwA may cause an increased sensitivity of the visual cortex to hypoxia and thus triggered attacks and increased fMRI responses
Faragó et al., 2019	18 MwA 33 MwoA 32 HC	Interictal	fMRI and DTI	Increased amplitude of activity fluctuations in networks of connectivity differences between migraine and HC in MwA group	Fundamental functional imaging differences in MwA compared to MwoA and HC
Russo et al., 2019	17 MwA (undifferentiated) 18 MwoA 15 HC	Interictal	fMRI	Increased BOLD response to trigeminal noxious stimulation in MwA in areas such as lingual gyrus, inferior parietal lobule and frontal gyri. Increased cerebellar activation in MwA	Dysfunctional correlations in pain modulating circuits and visual pathways in MwA with perhaps dysfunctional cerebellar inhibition on thalamic gating
Veréb et al., 2020	20 MwA (undifferentiated) 37 MwoA 32 HC	Interictal	fMRI	MwA shows more fluctuating connectivity within the salience network	Such changes may be related to cortical excitability and therefore aura
Coppola et al., 2021	35 MwA (20 visual, 15 additional sensory or speech; complex aura) 19 HC	Interictal	fMRI and DTI	Functional connectivity between DMN and dorsal attention system (DAS) is disrupted in both MwA groups compared to HC Thalamic diffusivity differs between visual aura and more complex aura and to HC The strength of DMN connectivity negatively correlates with bilateral thalamic DTI metrics in complex aura	Clinically heterogenous migraine aura subtypes may be associated with distinct structural and functional differences on brain imaging
Coppola et al., 2022	21 MwA 18 HC	Interictal	fMRI	Reduced interictal connectivity between default mode network (DMN) and left dorsal attention system (DAS) in MwA compared to HC. Following visual stimulation in MwA, improved connectivity between DAS and salience network and executive control network (ECN), with a negative correlation with monthly aura frequency	Stronger cognitive impact of visual stimulation on MwA compared to HC with reduced cognitive ability associated with increased frequency of aura
Gollion et al., 2022	21 MwA 18 HC	Interictal	fMRI	Strong functional coupling of right and left anterodorsal insulae with upper cerebellum in MwA only, not correlated with duration of diagnosis, frequency of aura or time since last aura	Involvement of brain regions thought to mediate autonomic function may explain some of the cardiovascular features of MwA
Park et al., 2022	11 MwA (undefined) 47 MwoA	Interictal	MRI, DTI and ASL	Global functional connectivity difference on ASL between two groups with no difference in structural parameters	Brain functional alterations in MwA and MwoA differ
Silvestro et al., 2022	20 complex MwA 20 MwA (visual) 20 MwoA	Interictal	fMRI	Complex MwA showed higher resting state functional connectivity of left lingual gyrus and of right anterior insula compared to migraine with typical visual aura and MwoA	Higher extrastriate brain changes may lead to aura propagation from simple visual aura to more complex aura phenotypes

MwA, migraine with aura; MwoA, migraine without aura; HC, healthy controls: fMRI, functional magnetic resonance imaging; BOLD, blood oxygen level dependant contrast; CSD, cortical spreading depression; ASL, arterial spin labeling; MRI, magnetic resonance imaging; VBM, voxel-based morphometry; DTI, diffusion tensor imaging; PAG, periaqueductal gray; DMN, default mode network; DAS, dorsal attention system; ECN, executive control network.

#### Ictal or peri-ictal studies

A key functional MRI (fMRI) study managed to image exertion-provoked migraine visual aura and the interictal period using an on/off visual stimulation paradigm. This study demonstrated fMRI signal in the occipital cortex (starting in V3a), correlating with the time of onset of the visual aura and the propagation correlating with the retinotopy of the visual percept (Hadjikhani et al., 2001). There was also a suggestion on the blood oxygen level dependent contrast (BOLD) of vasodilation followed by vasoconstriction, akin to that observed in CSD. This study for the first time used fMRI to visualize the aura itself, as well as the interictal phase in 3 subjects, and provided supportive imaging evidence for CSD mediating visual aura in the occipital cortex in this subject, and the propagation of this correlating with clinical symptoms. Similar findings had been previously observed using fMRI by Cao et al. (1999), with two captured auras (Cao et al., 1999).

Arngrim et al. (2017) were able to demonstrate differences in BOLD response over the visual cortex between positive and negative aura symptoms, suggesting a correlation between BOLD signal and clinical aura phenotype, and also suggested that occipital changes could occur bilaterally in bilateral aura. Hougaard et al. (2017a) imaged 16 migraine with visual aura ictally and demonstrated increased ictal connectivity between the left pons and somatosensory cortex, as well as between V5 and the middle frontal gyrus, providing possible links between brain areas involved in aura and those involved in headache. A further study by Arngrim et al. (2019) managed to capture 8 triggered aura attacks (using interictal exposure to hypoxia or sham gas) and image these with fMRI. The authors showed that hypoxia induced a larger BOLD response in the visual cortex of migraine with aura subjects compared to healthy controls in response to visual stimulation, and stipulated that migraine with aura may involve an inherent susceptibility of the visual cortex to hypoxia, triggered attacks and subsequent fMRI responses (Arngrim et al., 2019).

#### Interictal studies

A few studies have managed to use imaging to differentiate changes associated with different aura subtypes (pure visual aura compared to visual plus sensory or speech aura symptoms) (Coppola et al., 2018; Silvestro et al., 2022), and one has tried to distinguish positive and negative visual aura symptoms and correlate these with the BOLD response over the visual cortex (Arngrim et al., 2017). Coppola et al. (2021) suggested different degrees of altered thalamocortical cortical connectivity (thalamus and default mode network (DMN)) in migraine with visual aura compared to more complex aura (both being different to healthy controls interictally) and Silvestro et al. (2022) suggested that the insula is more involved in complex aura and is perhaps a cortical area of CSD propagation following visual cortex involvement. The insula region has also been suggested as being involved in mediating some of the cardiovascular autonomic features of migraine with aura (Gollion et al., 2022). Only one study has failed to demonstrate any interictal connectivity differences between migraine with aura and healthy controls in areas of interest including the occipital cortex (Hougaard et al., 2015b), however this study was a resting state study which did not involve any stimulation within the imaging paradigm. It is feasible that the abnormalities in brain function in migraine with aura are due to inherent network differences and/or altered neuronal excitability to sensory stimulation.

The use of these functional imaging techniques with different stimulation paradigms has in general suggested ictal and interictal differences between migraine with and without aura and healthy controls in brain function, and unsurprisingly changes involving areas like visual and somatosensory cortices. Additional cortical, thalamic and insular involvement suggests that aura involves or causes more widespread brain dysfunction in areas involved in sensory, executive and limbic processing. The differences in imaging methodologies and acquisition parameters, paradigms, subject selection, image processing and analyses make the reproducibility of fMRI studies a challenge in any field. The clinical heterogeneity of migraine contributes to the variance in imaging data and difficulties with producing consistent findings. There is however suggestion of alterations in ictal and interictal brain function in key cortical and subcortical regions in migraine with aura. Use of more advanced imaging methodologies and paradigms may enable further characterization of different aura types with such imaging, as a means to producing ictal and interictal biomarkers for different aura subtypes.

### Other imaging modalities

In addition to perfusion and fMRI approaches, the use of other imaging methodologies has been increasing in migraine research as availability and expertise has advanced in the field. These have included MR spectroscopy (MRS), magnetoencephalography (MEG) and combination approaches using positron emission tomography (PET) and MR together.

Ligand-based PET and MR approaches interictally have suggested glial activation in patients with migraine and recent aura attacks in the visual cortex and Broca’s area. Changes have also been reported in somatosensory cortex and thalamus, and frontal and orbitofrontal cortex, with a positive association between the signal strength and baseline aura frequency (Albrecht et al., 2019). Occipital meningeal and bone involvement (Hadjikhani et al., 2020) has been observed, with suggestions of possible links between CSD and trigeminal pain. These studies have again suggested wider cortical involvement beyond the visual cortex and involvement of the thalamus in aura mechanisms. The role for the meninges and the possibility of an altered blood brain barrier in migraine with (Hougaard et al., 2017a; Hadjikhani et al., 2020) and without aura (Amin et al., 2017) has been disputed between different studies.

Spectroscopy studies have suggested a possible contribution of mitochondrial dysfunction in migraine, possibly secondary to the brain cortical excitability change as a mechanism of predisposition to CSD (Barbiroli et al., 1992; Sarchielli et al., 2005; Sándor et al., 2005; Arngrim et al., 2016; Zielman et al., 2017). Whilst the studies have produced heterogeneous findings, it has been postulated that identified energy disturbances may be related to the clinical phenotype, and alterations have more frequently been demonstrated in migraine with aura patients compared to those without aura (Sarchielli et al., 2005; Sándor et al., 2005). In migraine with aura, an elevated lactate peak is present before and after visual stimulation in migraine with visual aura, but only peaks during visual stimulation in more complex aura, suggesting that pure visual aura may involve more lactate transporter system dysfunction compared to other aura subtypes (Sándor et al., 2005). During photic stimulation in patients with migraine with aura, the decrease in N-acetylaspartate (NAA), as a marker of mitochondrial dysfunction, suggests a less efficient mitochondrial functioning state in these patients (Sarchielli et al., 2005). Increased glutamate levels in the visual cortex interictally have also been demonstrated in migraine with aura patients (Zielman et al., 2017), and an abnormal reduction of response from the occipital cortex to interventions that change cortical excitability (transcranial direct current stimulation) has also been reported (Siniatchkin et al., 2012).

These advanced imaging modalities have supported wider cortical involvement in aura beyond the visual cortex and raised the possibility of interictal altered mitochondrial function and increased cortical excitability (without a change in inhibitory mechanisms) in migraine with aura. Larger studies are required to evaluate these findings and their reproducibility and clinical translation more systematically in the future. Some of these studies are summarized in Table 4.

**TABLE 4:** A summary of studies using other advanced imaging techniques in migraine with typical aura.

References	Subjects	Imaging phase	Imaging modality	Main findings	Translation of findings
Barbiroli et al., 1992	12 MwA	Interictal	MRS	Low phosphocreatine content in all subjects, accompanied by high adenosine diphosphate concentration, a high percentage of V/Vmax (adenosine triphosphate), and a low phosphorylation potential. 75% had abnormal muscle mitochondrial function without clinical signs	MwA involves an unstable metabolic state, and may be a feature of a more systemic mitochondrial disease
Bowyer et al., 2001	12 MwA 6 HC	Ictal (8 visually induced, 4 spontaneous)	MEG	Complex direct current MEG shifts in spontaneous and visually induced migraine patients, but not in controls. Multiple cortical areas activated in spontaneous and visually induced aura. Activation only observed in the primary visual cortex in HC	A spreading, depression-like neuroelectric event occurs during migraine aura that can be spontaneous or visually triggered in widespread regions of hyperexcitable occipital cortex
Sándor et al., 2005	10 MwA (5 visual and 5 visual plus speech or sensory) 11 HC	Interictal	MRS	In complex aura, lactate increased only during prolonged visual stimulation in visual cortex; in visual aura, lactate high in visual cortex, without further increase during stimulation	Abnormal cortical metabolism during visual stimulation in complex aura patients and aura may be caused by mitochondrial dysfunction
Sarchielli et al., 2005	22 MwA 22 MwoA 10 HC	Interictal	MRS	Reduced NAA signal in MwA group compared to others in response to photic stimulation with slight lactate increase	Differences in mitochondrial function in MwA
Arngrim et al., 2016	15 MwA 14 HC	Interictal during hypoxia exposure (7 subjects may have had aura triggered)	MRS and MRA	Hypoxia did not change glutamate concentration in the visual cortex compared to sham, but increased lactate concentration and circumference of cranial arteries. No difference in the metabolic or vascular responses to hypoxia between migraine patients and controls.	Hypoxia induced migraine-like attacks with and without aura and dilated the cranial arteries in patients with MwA. Hypoxia-induced attacks were not associated with altered concentration of glutamate or other metabolites.
Zielman et al., 2017	23 MwA 27 MwoA 24 HC	Interictal	MRS	Increased interictal cortical glutamate in MwoA	MwoA is associated with cortical hyperexcitability
Stærmose et al., 2019	14 MwA (infrequent migraine) 16 HC	Interictal	MRS	No difference in cortical GABA between MwA and HC in occipital or somatosensory cortices	Unclear role of inhibitory GABAergic system in mediating aura
Albrecht et al., 2019	13 MwA 16 HC	Interictal	PET and MRI	Increased neuroimmune activation or neuroinflammatory activity in MwA in areas like somatosensory, insular and visual cortices and thalami	CSD may be associated with glial activation and neuroinflammation
Hadjikhani et al., 2020	11 MwA (visual)	Within one month of migraine with visual aura	PET and MRI	Strong and persistent extra-axial inflammatory signal in meninges and calvarial bone overlying occipital lobe in migraine with visual aura	CSD may produce meningeal inflammation and lead to marrow changes

MwA, migraine with aura; MwoA, migraine without aura; HC, healthy controls; MRS, magnetic resonance spectroscopy; MEG, magnetoencephalography; NAA, N = acetylaspartate; MRA, magnetic resonance angiography; GABA, gamma-aminobutyric acid; PET, positron emission tomography; CSD, cortical spreading depression.

## Discussion

The use of increasingly advanced structural and functional imaging techniques has allowed the study of migraine in humans in ways that were not possible before. In particular, the ictal imaging of aura both clinically and using functional imaging methods, has provided a feasible imaging correlate for CSD mechanisms observed in preclinical models. The increasing use of perfusion-based imaging modalities in clinical practice, largely through stroke services, may provide a unique opportunity to capture aura ictally more frequently than we have been able to in the past, and provide a means of diagnostic differentiation, as well as potential mechanistic insights into aura mediation.

The imaging studies using different structural and functional imaging modalities have compared both migraine with and without aura to each other, and at times have evaluated migraine with aura alone or in comparison to healthy controls. Clearly, there are limitations to studies in which there is no comparison group, and in those that do not include a migraine without aura group. It can be difficult to make inferences about the biology of aura from such studies, but they can provide supportive evidence of differences between the migraine with aura brain in general, and in comparison to healthy controls.

Overall, the studies seem to suggest that aura is likely mediated by widespread brain dysfunction in areas involving, but not limited to, visual cortex, other cortical areas and thalamus, therefore suggesting altered cortical hyperexcitability and sensory sensitivity. The interictal predisposition to aura among sufferers, and differences in cortical excitability in response to sensory stimulation compared to healthy controls and migraine without aura, are a likely result of genetic influences and interictal alterations in resting brain structure and function. Pure visual aura and visual aura with other sensory or speech symptoms may involve different functional reorganization of brain networks and mitochondrial dysfunction.

The differences in study size, subject recruitment and screening, imaging modalities, scanner strength and analysis methods, are challenges in the study of any condition. In migraine, baseline headache frequency, disease duration, medication use, clinical heterogeneity, genetics and phase of the migraine cycle and comorbid psychological and psychiatric disorders, are just some of the additional factors that contribute to variance amongst imaging data sets. The definition of aura and which subjects are diagnosed with migraine with aura is another area which may not be as clear as one may think in clinical practice. The difficulties with studying aura ictally contribute to these challenges. The general lack of consistent ability to provoke aura in experimental settings, using natural trigger factors (Hougaard et al., 2013) and using pharmacological migraine provocation agents such as nitroglycerin (NTG) (Christiansen et al., 1999), calcitonin gene-related peptide (CGRP) (Hansen et al., 2008, 2010) and sildenafil (Butt et al., 2022), despite these being potent experimental triggers for migraine headache and other associated migraine symptoms, suggest that aura and headache mechanisms are perhaps distinct in migraine. The majority of imaging studies conducted to evaluate aura, or the differences between migraine with and without aura, have been performed interictally in the absence of symptoms.

The differences in baseline stroke risk, heritability of migraine, treatment response to acute and preventive migraine medication and possible changes in structural and functional imaging, support fundamental biological differences between migraine with and without aura (Hansen and Charles, 2019). The heterogeneity in aura phenotype, timing and duration between and within patients with migraine with aura (Viana et al., 2017) is also interesting and supports the theory that migraine is a heterogenous umbrella disorder, likely involving various neurobiological mechanisms, not all of which are unanimously implicated in all sufferers. Whilst migraine aura with visual components is the most common phenotype, the individuals that also experience speech and sensory symptoms may have different genetic and neurobiological mechanisms contributing to the additional aura phenotype. Such differences amongst migraineurs may also explain the differences in treatment efficacy amongst different migraine preventive medications in patients with migraine, where even with the newly emerged CGRP antibodies, a significant proportion of sufferers will not respond to therapy (Cullum et al., 2022). This disease and treatment-related heterogeneity is poorly understood and makes the individualization of therapy challenging for physicians. At present, owing to lack of systematic and large randomized clinical trials in this area, management options for migraine with and without aura are shared. Agents such as ketamine (Kaube et al., 2000; Afridi et al., 2013), aspirin (Anoaica et al., 2014; Turk et al., 2017), amiloride (Holland et al., 2012) and levetiracetam (Brighina et al., 2006) have been used in clinical case series or unblinded studies of aura with some suggestions of efficacy, but not enough evidence to support their wide scale clinical use. There is a suggestion that triptans are less useful acutely in migraine with aura attacks compared to those without aura (Hansen et al., 2015), and that single pulse transcranial magnetic stimulation is more useful in treating migraine with aura compared to migraine without aura (Lipton et al., 2010; Lan et al., 2017). Migraine preventive drugs tend to lower the threshold for CSD (and CSD has been used as a preclinical model to test drug efficacy) or lower the frequency of CSD’s, suggesting there may be a link between CSD and trigeminal nociception (Ayata et al., 2006), but it is important to remember that many traditionally used migraine drugs have broad mechanisms of action unspecific for migraine, so may alter CSD thresholds in ways unrelated to their anti-migraine effect. In clinical practice, the use of commonly used migraine preventives is rarely sufficient for the management of problematic aura symptoms in our experience.

There are several other questions and challenges that aura poses. The possible mediation of asymptomatic aura (although this would clearly not meet clinical criteria for an aura diagnosis) within the brain in some individuals, perhaps propagating in silent or less eloquent areas of cortex (Karsan et al., 2018; Hadjikhani and Vincent, 2021), how and why aura starts in visual cortex and can spread to other cortical areas, why some attacks are not associated with aura symptoms despite the presumed same cortical areas being involved across attacks, and the potential links between CSD and trigeminal nociception, are just some of these. Several associated migraine symptoms distinct from aura are also mediated via cerebral cortex and there is a lack of clinical or radiological means of differentiating these clinically and neurobiologically from aura. These include reversible alterations in speech (Schwedt et al., 2019) and cognition (Vincent and Hadjikhani, 2007), which can occur in the premonitory phase in the lead up to a migraine attack (Karsan and Goadsby, 2018), as can aura. In our practice, we would not usually classify transient non-specific visual blurring or non-aphasic or non-dysarthric speech symptoms associated with a migraine attack as typical aura symptoms, but more as premonitory or premonitory-like symptoms if occurring preceding or during pain respectively, or postdrome symptoms if occurring after headache resolution. This is largely because they are not necessarily localizable to one area of cortex, lack anatomical lateralisability do not necessarily spread in the same way other aura symptoms do, and could not be classified as ‘positive’. These are all characteristics ICHD3 defines as being consistent with aura and some of which have to be met to make the diagnosis (Headache Classification Committee of the International Headache Society [IHS], 2018). However, it is feasible that in some studies, these symptoms could be included in imaging studies, and the potential symptomatic differences between such symptoms and true aura is an area that should be explored in further iterations of the International Classification of Headache Disorders. As well as contributing to data variance in imaging studies, such symptoms may pose diagnostic and classification challenges for clinicians when occurring before or during headache and may prompt investigations for secondary causes. Systematic and classification-based distinction of these from aura, is in our opinion, an important area going forwards.

## Conclusion

Migraine with aura is a complex heterogenous condition, likely mediated by widespread brain dysfunction ictally and interictally in areas involving but not limited to visual cortex, and also involving other cortical areas and thalamus. Neuroimaging, with increasingly advanced clinically available techniques being used largely due to advances in hyperacute stroke management, and novel and emerging research methodologies, have greatly contributed to our understanding of the neurobiological bases of migraine and migraine aura. However, differences in scanner strengths and imaging acquisition, imaging modalities, patient recruitment methods, sample sizes, aura diagnosis and analysis methods, among other issues make reproducibility of imaging findings across studies and centers challenging. Outside of the scope of this review, there have also been several case reports and case series reporting of imaging changes associated with aura. The brain changes discussed in this review in general suggest that there are distinct changes in migraine with aura compared to migraine without aura and healthy controls both ictally and interictally and allude to a state of cortical hyperexcitability and altered sensory processing in migraine with aura, likely mediated by a combination of structural and functional brain changes. These findings support neurophysiological literature in this area. The interictal predisposition to aura and differences in cortical excitability in response to sensory stimulation are probably a result of genetic influences and interictal alterations in resting brain structure and function. Pure visual aura and visual aura with other sensory or speech symptoms may involve different functional reorganization of brain networks and mitochondrial dysfunction. Larger more systematic population-based imaging studies with larger cohorts of subjects with different aura subgroup analyses are required to characterize these findings further and shed further light on the possible neurobiological differences in different aura presentations. Given the visual predominance in aura symptoms even in those that also develop other symptoms, a genetic basis to predisposition to occipital CSD via altered cortical excitability and perhaps mitochondrial mechanisms seem most likely, but the reasons that the majority of attacks do not involve aura, the links between CSD and trigeminal pain and how CSD propagates to different cortical areas from occipital cortex and why are questions that remain unanswered. Going forwards, developing provocation models of aura to allow ictal imaging, and advances in routine clinical imaging methodologies for the acute imaging of aura, will be key ways to develop such understandings and contribute to the development of imaging biomarkers for aura, increase the use of diagnostic imaging acutely, and allow personalization of migraine therapeutics. This is an area that all clinicians that treat migraine would find attractive, as despite many therapeutic advances in the field, prediction of treatment response and specific treatment of problematic and disabling aura are areas that need further work.

## Author contributions

NK and ES wrote the manuscript following review of the literature. NK made the tables. PG was involved in senior review of the manuscript. All authors have reviewed the manuscript prior to final submission.
